# Supplementary figures and images for: Effect of Vitamin-D-Enriched Edible Mushrooms on Vitamin D Status, Bone Health and Expression of CYP2R1, CYP27B1 and VDR Gene in Wistar Rats
Source: J Fungi (Basel). 2022 Aug 17;8(8):864. doi: 10.3390/jof8080864 (PMC9409838; doi:10.3390/jof8080864)

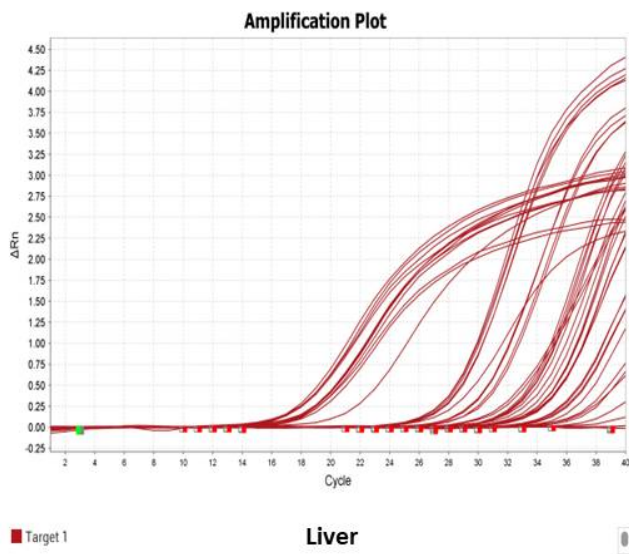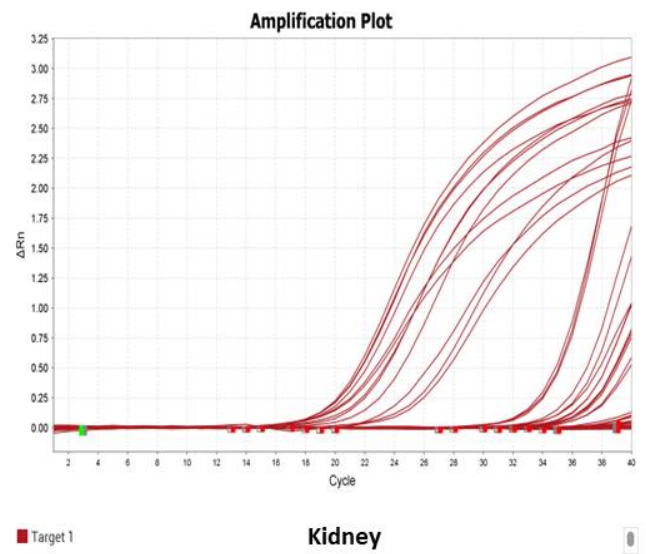

**Figure S1.** Real-time PCR amplification plots of CYP2R1, CYP27B1 and VDR gene in liver and kidney tissue.

Supplement: Supplementary file 1 [file jof-08-00864-s001.zip › jof-1855936-supplementary.pdf]
